# Politics and the State Medical Association

**Published:** 1907-12

**Authors:** 


### EDITORIAL DEPARTMENT.

### POLITICS AND THE STATE MEDICAL ASSOCIATION.

The effort to drag the State Association into politics and make the recent bungling and unpopular medical legislation,—the One Board Bill,—an issue in the next election, is greatly to be deprecated. The Association should be kept out of it. The effect of such policy will be disastrous; the older and more quiet and conservative members will just stay away from meetings and lose all interest in the affairs of the organization.

In organizing on the present plan, the majority of the membership disfranchised themselves and put all power in the hands of a few—say 2 per cent—who constitute the House of Delegates. That the House of Delegates, consisting of one delegate from each county society, does not always represent the sentiment of the profession, I am well satisfied. Especially in the matter of the committee inviting the irregulars to affiliation, and homologating with them on a mixed board, I have abundant reason to believe that they have gone counter to the wishes and sentiment of the majority.

And now comes a newspaper announcement that one "high in medical councils"—doubtless one of those gentlemen who are responsible for the obnoxious one board—has committed the whole Association—*nolens volens*—to a policy which I *know* is foreign to the general sentiment of the membership.

In the Associated Press dispatches, November 17th, there appeared a statement from the San Antonio correspondent, upon the authority of "one high in medical councils," to the effect that the Texas State Medical Association, resenting the alleged opposition of the Attorney General to the One Board Bill, would "boycott" him, and throw their whole influence against his re-election, and in favor of Senator Looney (the Senator who championed the bill and advocated breaking down all barriers between the regulars and the irregulars,—putting all the "brethren" on an equal footing).

I believe that if the rank and file (the great majority) could be heard, it would be found that they do not endorse the action of the committee in inviting and insisting upon fraternizing with those whom we have always regarded and denounced as irregulars. I believe they would repudiate the policy to which the Association, unheard, has been committed. And I denounce as unwarranted and untrue the statement that the Association will boycott Mr. Davidson. No one is authorized to speak for the Association. I am not advocating Mr. Davidson (I am not even acquainted with him), nor inveighing against Mr. Looney. I am condemning the effort to make political capital out of the One Board Bill—to be used against one candidate and for the other; but it is only just to say that Mr. Davidson had nothing whatever to do with the bill, and that the State Medical Association will not boycott him. I know the sentiment pretty well, and he will not be made a scapegoat by the Association to be sacrificed in the interest of his opponent. Governor Campbell assumes all responsibility for opposition to the bill, and would have vetoed it but for changes made in it by Mr. Hawkins, the Office Assistant Attorney General, at his, Mr. Campbell's, request. Davidson never saw the bill.

I do not know who the "man high in medical councils" is, but I can guess. He should be sat down on. He has misrepresented the Association. He has no right to commit them to anything. This declaration followed immediately on the heels of a meeting of the San Antonio (Fifth) District Medical Society, at which Mr. Looney was present and spoke.

The unification idea emanated from the great medico-political machine at Chicago—the Octopus (*Journal A. M. A.*)—and the skeleton of a One Board Bill was furnished to each State Medical Association. That the House of Delegates is dominated by the great machine goes without saying,—and our Committee on Legislation sacrificed every principle of professional ethics and pride—at the dictates really, of an apostate homeopath, the Power behind the Octopus, *Simmons*.

